# Manipulating Entanglement Dynamics in Dephased Interacting Qubits Using a Radiation Field

**DOI:** 10.3390/e27070673

**Published:** 2025-06-24

**Authors:** Omar Qisieh, Rahma Abdelmagid, Gehad Sadiek

**Affiliations:** 1 Department of Applied Physics and Astronomy, College of Sciences, University of Sharjah, University City, Sharjah 27272, United Arab Emirates; u21105536@sharjah.ac.ae (O.Q.); u22104927@sharjah.ac.ae (R.A.); 2 Department of Physics, Ain Shams University, Cairo 11566, Egypt

**Keywords:** Tavis–Cummings model, dephasing environments, open quantum systems, quantum information, Ising interaction, isotropic interaction, anisotropic interaction

## Abstract

We study the entanglement dynamics of a pair of non-identical interacting atoms (qubits) coupled off-resonance to a single-mode cavity radiation field and exposed to dephasing environments. The qubits are studied starting from various initial states that are disentangled from an initially coherent field. The system models the basic building units of quantum information processing (QIP) platforms under the realistic considerations of asymmetry and external environmental influences. We investigate how introducing a radiation field alters the system’s entanglement dynamics in the presence of dephasing environments, and how it impacts the effects of the dephasing environments themselves. The work examines the problem under various settings of inter-qubit interactions, which are now experimentally controllable in some of the newly engineered artificial qubit systems. We illustrate that only upon introducing the radiation field, the system suffers a terminal disentanglement (followed by no revivals) in a finite time. This behavior is exacerbated when the atoms’ interaction with the field is stronger. Moreover, the effects of the field’s intensity and the atoms’ detunings are vastly sensitive to the choice of the initial state. We also demonstrate that the closer the atoms’ transition frequencies are to resonance with the field, the more pronounced are the effects of strengthening the independent dephasing environments corresponding to some initial states. Those states also suffered a greater reduction in entanglement content when the qubits with stronger atom–field interaction strength were influenced by a stronger independent dephasing environment. In addition, we examined the ability of the correlated dephasing environment to induce a noise-enhanced efficiency in the presence of an external radiation field. We showed that the radiation field could play a decisive role in enabling or restricting noise-enhanced efficiency, but one that is also highly sensitive to the system’s initial state.

## 1. Introduction

The interactions between atoms and radiation fields have substantial practical implementations in the field of quantum information processing. In 1963, the Jaynes–Cummings model offered the first ever exact quantum mechanical treatment for the interaction between a two-level atom and a quantized radiation field [1,2]. Subsequently, the long-proposed problem (by Dicke [3]) of a non-interacting multi-atom system coupled to a common coherent radiation field was successfully solved in the famous Tavis–Cummings model [4]. With the advent of the field of quantum information processing, these models served as stepping stones that physicists frequently referred to for analyzing more intricate quantum systems of practical interest in the field.

Subsequent research extensively leveraged these foundational models to explore more realistic scenarios. For instance, a study undertaken by Eberly and Yu [5] revealed that entangled qubits (two-level atoms) undergo sudden entanglement loss when they are independently coupled to pure vacuum noise. They named this phenomenon entanglement sudden death (ESD) and demonstrated that it occurs in a finite time period. In the same context, the sudden emergence of entanglement between two initially disentangled atoms was observed by Ficek and Tanaś and was named entanglement sudden birth (ESB) [6].

The emergence of entanglement between atoms that are not directly interacting has garnered theoretical and experimental interest. For example, investigations into entanglement sharing in a system comprising two non-interacting identical atoms coupled with resonant radiation fields in the Fock state or coherent state were carried out [7,8]. However, recent developments in the field led to newly engineered qubits that are experimentally accessible (such as superconducting circuits and quantum dots) and which enjoy highly customizable qubit–qubit interactions [9,10,11]. This customization offered a new approach for controlling the system dynamics, namely, through manipulating its degree of anisotropy [12]. Given the role the degree of anisotropy plays in determining the qubits’ suitable physical manifestation for a specific QIP architecture, a more thorough investigation of the different possible degrees of anisotropy is in order. For example, the superconducting qubits experience anisotropic interactions [13], while the quantum dots are generally isotropic [14,15].

With recent advancements in the field that allow the utilization of hybrid qubit structures in the same cavity and superconducting resonators, studying the behavior of entanglement in non-identical (asymmetric) qubit systems has garnered great research interest [16,17,18,19,20,21]. A thorough investigation into the influences of asymmetry parameters has the potential of introducing new regimes of effective control over the entanglement dynamics compared to symmetric systems, for it facilitates the control of two-qubit gates (exchange gates), reduces decoherence effects, and enhances one-direction steerability [22,23,24,25]. Remarkably, the generation of entanglement in a hybrid asymmetric qubit system can be achieved using a microwave cavity, even when the sizes of the qubits differ by orders of magnitude, reaching $10^{3}$ [26]. On the other hand, the inclusion of dissipation and dephasing effects due to interactions with the external environment in the analysis is a necessary practical consideration that has profound impacts on the system’s dynamics. In particular, understanding the dynamics of the system under such environmental influences and its relation to the duration for which the system preserves coherence is essential to ensure the effective achievement of quantum information processing tasks [27,28,29,30,31]. In this context, the ESD caused by the interaction with a dephasing environment was discussed within the teleportation scheme, where the qubit interacts individually with the environment [32]. This highlights the importance of accounting for environmental effects on each element when aiming to achieve useful QIP. The influence of independent dephasing and dissipative channels on systems of two non-interacting qubits was also studied in [28], where the entanglement dynamics of qubits initially in a mixed entangled state were analyzed. ESD also occurs when the qubits are subjected to collective depolarizing channels, which are considered a type of dephasing environment [33]. It is important to note that in these studies, the qubits were assumed to be identical, non-interacting, and initially entangled.

In a phenomenon dubbed noise-enhanced efficiency, the literature has shown that the presence of a noisy environment can cause an increase in the entanglement content between the qubits or even a deceleration of decoherence between them [34,35]. The origin of this phenomenon is driven by multiple factors, including the induction of non-local correlations across subsystems and avoidance of quantum localization. In particular, interference effects in quantum states can confine the system to a specific range of states, leading to quantum localization in which state changes become restricted and thereby impeding the overall dynamics [36,37,38]. On the other hand, Caruso et al. [35] found that the enhanced efficiency follows from how the dephasing noise reshapes the interference patterns within quantum networks. More precisely, when a controlled amount of dephasing noise is introduced to the system, it randomizes the relative phases. This diminishes the destructive interference that may occur as the quantum information propagates along different coherent pathways and limits the effective transmission of the quantum state. Moreover, Bruan and Benatti et al. [34,39] showed that two non-interacting initially disentangled systems can build up quantum correlations with the aid of coupling with a common environment (noise source), which enables indirect or auxiliary interactions between them. The entanglement is then generated when the bath state correlates operators associated with each qubit. Furthermore, Gillard et al. [40] showed that there is a critical point that defines whether noise-enhancement efficiency will occur or not. This critical point depends on the level of noise and the initial state of the quantum system, which, consequently, determines whether the entropy will increase, remain constant, or decrease when the noise is increased.

However, interesting behavior of the qubits systems happens when they interact with each other. Das and Agarwal [41] examined two XX-interacting qubits coupled to dissipative and dephasing environments and studied the evolution of the system’s entanglement. They found that the interplay with such environments leads to periodic ESD and ESB (bright and dark periods), and that the correlated environments can delay the disentanglement of the qubits. Furthermore, a model of XYZ-interacting qubits coupled with a thermal reservoir was investigated [42]. However, only a maximally entangled initial state was considered, and the environment was modeled as a bosonic bath. Li et al. [43] also studied collapse and revival patterns in two central spin systems interacting with independent spin baths, starting from initially disentangled qubits. They demonstrated that the interaction with the spin bath induces entanglement between the qubits. Moreover, the emergence of entanglement between two strongly coupled qubits via dipole–dipole interaction was studied in [44]. Their system architecture enabled the creation of long-lived entanglement between initially disentangled qubits, where two types of reservoirs were considered: a separate dephasing reservoir for each qubit and a collective radiative reservoir shared by both. So far, the literature has yet to provide a detailed analysis of how the anisotropy of the inter-qubit interaction may alter or limit the impact of environmental effects over the various potential settings of the qubits’ initial states.

In this paper, we delve into the dynamics of entanglement in a system of two interacting qubits coupled to an initially coherent single-mode radiation field as well as dephasing environments. The dephasing effects are modeled by two different types of environments: a correlated dephasing environment that influences both qubits simultaneously, and two separate independent dephasing environments that influence each qubit separately. The work investigates three different levels of anisotropy of the inter-qubit coupling over ranges of atom–field interactions, qubits’ natural transition frequencies, and field’s intensity and frequency mode with simultaneous inclusion of dephasing environmental influence. The work demonstrates the interplay between these different factors, providing an understanding of the behavior of the basic building units of QIP systems and paving the way for their utilization to create more stable QIP protocols that are more persistent under environmental effects. In a previous work [12], we investigated the same system of qubits under the effect of the same environments but in the absence of any radiation field, which plays a crucial role in contemporary technologies implemented in the control and reading of qubit system states [45,46,47].

In the following section, we present our model, including the Hamiltonian and the mathematical description of the dephasing environments, trailed by an introduction to the master equation and its solution and the used measure of entanglement. In Section 3, we investigate how the presence of the radiation field alters the dynamics of entanglement corresponding to different settings of the system internal parameters and a fixed external environmental effect. Section 4 examines how different settings of the environment will change the entanglement’s evolution, and shows how the presence of the field can be used to control those changes. Finally, we summarize our findings and conclude in Section 5.

## 2. Mathematical Formulation

### 2.1. The Model

We study a system of two two-level interacting qubits coupled to a radiation field in an open system that is susceptible to dephasing environments. The two levels are indicated by $|g_{i}\rangle$ (ground state) and $|e_{i}\rangle$ (excited state), where i=1,2 refer to the first and second qubits, respectively. The work focuses on three classes of systems: a partially anisotropic system (XYZ Model), a maximally anisotropic system (Ising model), and an isotropic system (XXX Model). The Hamiltonian of the system is given by (ℏ=1)

(1) $$H=\Omega a^{†}a+\sum_{i=1}^{2}\omega_{i}S_{i}^{z}+\sum_{i=1}^{2}\lambda_{i}\left(aS_{i}^{+}+a^{†}S_{i}^{-}\right)+J\left(\frac{1+\gamma }{2}S_{1}^{x}S_{2}^{x}+\frac{1-\gamma }{2}S_{1}^{y}S_{2}^{y}+\delta S_{1}^{z}S_{2}^{z}\right).$$

In the above equation, the first two terms represent the free energies of the radiation field and the two interacting qubits, respectively, where Ω is the frequency of the field and $\omega_{i}$ is the natural transition frequency of the ith qubit. Here, $a^{†}$ and *a* are, respectively, the field’s creation and annihilation operators, and $S_{i}^{\alpha }$ denotes the α component of the ith qubit spin operator. The interaction of the ith qubit with the radiation field is determined by the coupling constant $\lambda_{i}$, while the strength of the interaction among the qubits themselves is governed by the exchange energy *J*. The anisotropy of the inter-qubit interaction, on the other hand, is controlled by the parameters δ and γ. In particular, the aforementioned three degrees of anisotropy are going to be investigated by setting the parameter pair (γ,δ) to (1/2,1) for the XYZ model, (1,0) for the Ising model, and (0,1/2) for the XXX model. To simplify the analysis, a basis that conserves the number of excitations is chosen to represent the composite system:

(2) $$\left\{|e_{1},e_{2},n\rangle ,\;|e_{1},g_{2},n+1\rangle ,\;|g_{1},e_{2},n+1\rangle ,\;|g_{1},g_{2},n+2\rangle \right\},$$

where |n〉 is the field’s Fock state. In this basis, the matrix representation of the Hamiltonian (H) reads

(3) $$H=\frac{1}{4}\left(\begin{array}{cccc} 4\Omega n+2\tau +\delta J & 4\lambda_{2}\sqrt{n+1} & 4\lambda_{1}\sqrt{n+1} & \gamma J\\ 4\lambda_{2}\sqrt{n+1} & 4\Omega (n+1)+2\epsilon -\delta J & J & 4\lambda_{1}\sqrt{n+2}\\ 4\lambda_{1}\sqrt{n+1} & J & 4\Omega (n+1)-2\epsilon -\delta J & 4\lambda_{2}\sqrt{n+2}\\ \gamma J & 4\lambda_{1}\sqrt{n+2} & 4\lambda_{2}\sqrt{n+2} & 4\Omega (n+2)-2\tau +\delta J \end{array}\right),$$

where $\tau =\omega_{1}+\omega_{2}$ and $\epsilon =\omega_{1}-\omega_{2}$.

In the context of an open system, the general framework of master equations can be used to incorporate environmental influences on the dynamics of the system. More precisely, the evolution of the density operator (ρ) is governed by the Lindblad master equation of motion, which describes the evolution of the statistical state of the system [48,49,50]:

(4) $$\dot{\rho }\left(t\right)=-i\left[H,\rho \right]+D_{\rho },$$

where, in the Lindblad form,

(5) $$D_{\rho }=\frac{1}{2}\sum_{j}\sum_{i}\left\{\left[L_{i}^{\left(j\right)}\rho ,L_{i}^{(j)†}\right]+\left[L_{i}^{\left(j\right)},\rho L_{i}^{(j)†}\right]\right\},$$

and the Lindbladian $L_{i}^{\left(j\right)}$ represents the operation of the jth environment on the ith subsystem. The first term in Equation (4) represents the unitary elements of the time evolution due to the system’s internal constituents, while the second term introduces the non-unitary contribution of the environments. In the current work, the environments are assumed to be acting on the qubits only, where each qubit is influenced by a separate independent dephasing environment ($L_{i}^{\left(i\right)}=\sqrt{2{\bar{\Gamma }}_{i}}S_{i}^{z}$) together with a common correlated dephasing environment acting on both qubits at once. Consequently, the master equation will assume the form

(6) $$\begin{array}{ccc} \dot{\rho }\left(t\right) & = & -i\left[H,\rho \right]-\sum_{i=1}^{2}\Gamma_{i}\left(S_{i}^{z}S_{i}^{z}\rho +\rho S_{i}^{z}S_{i}^{z}-2S_{i}^{z}\rho S_{i}^{z}\right)\\ & & -2\Gamma_{0}\left(S_{1}^{z}S_{2}^{z}\rho +\rho S_{1}^{z}S_{2}^{z}-S_{1}^{z}\rho S_{2}^{z}-S_{2}^{z}\rho S_{1}^{z}\right), \end{array}$$

where $\left\{\Gamma_{i}\right\}$ are the environments’ dephasing rates. Working in Liouville space, the governing equation can be written as a linear differential equation in terms of the density operator [50]:

(7) $$\dot{\rho }\left(t\right)=\left({\hat{L}}^{H}+{\hat{L}}^{D}\right)\rho =\hat{L}\rho ,$$

where ${\hat{L}}^{H}$ and ${\hat{L}}^{D}$ are the corresponding contributions of the first and second terms in Equation (4), and $\hat{L}$ is the total contribution known as the Liouville superoperator. By diagonalizing the Liouville superoperator, the density operator at any time *t* is given by

(8) $$\rho \left(t\right)=\sum_{i}c_{i}\varrho_{i}e^{\nu_{i}t},$$

where $c_{i}$ are coefficients determined by the initial setup of the system at t=0, $\varrho_{i}$ are the eigenvectors of $\hat{L}$, and $\nu_{i}$ are the corresponding eigenvalues. By tracing out the field, one can obtain the reduced density matrix of the two qubits as

(9) $$\rho_{red}\left(t\right)={\mathrm{Tr}}_{\mathrm{field}}\;\rho \left(t\right)=\sum_{l}\langle l|\rho \left(t\right)|l\rangle .$$

To exemplify the system behavior, we will consider three different initial states of the two-atom system: a pure atomic system in a disentangled state given by

(10) $${|\psi \rangle }_{D}=\left(|e_{1},e_{2}\rangle +|e_{1},g_{2}\rangle +|g_{1},e_{2}\rangle +|g_{1},g_{2}\rangle \right)/2,$$

a pure atomic system in the correlated Bell state

(11) $${|\psi \rangle }_{Bc}=\left(|e_{1},e_{2}\rangle +|g_{1},g_{2}\rangle \right)/\sqrt{2},$$

and a mixed atomic system in a Werner state described by the density operator

(12) $$\rho_{Wr}=\left(a|e_{1},e_{2}\rangle \langle e_{1},e_{2}\left|+d\right|g_{1},g_{2}\rangle \langle g_{1},g_{2}\left|+2|\phi \rangle \langle \phi \right|\right)/3,$$

where *a* and *d* are independent parameters that shape the nature of the initial state and satisfy d=1−a. The state |ϕ〉 is given by

(13) $$|\phi \rangle =\left(|e_{1},g_{2}\rangle +e^{i\pi /4}|g_{1},e_{2}\rangle \right)/\sqrt{2}.$$

As for the radiation field, it is assumed to be initially in a coherent state (|α〉) which is disentangled from the two-qubit system:

(14) $$|\alpha \rangle =\sum_{n}Q_{n}|n\rangle ,\;\;Q_{n}=\frac{\alpha^{n}}{\sqrt{n!}}\exp\left(-\frac{{\left|\alpha \right|}^{2}}{2}\right),$$

where ${\left|\alpha \right|}^{2}=\bar{n}$ denotes the mean number of photons. Thus, for a two-qubit system starting from the statistical mixture $\sum_{i}w_{i}|\varphi_{i}\rangle \langle \varphi_{i}|$, the initial density operator of the composite system is given by

(15) $$\rho \left(0\right)=\sum_{i}w_{i}\left(|\varphi_{i}\rangle ⊗|\alpha \rangle \right)\left(\langle \varphi_{i}\left|⊗\langle \alpha \right|\right).$$

### 2.2. Quantifying Entanglement

The entanglement between a pair of qubits can be quantified through the concurrence $C(\rho_{red})$ introduced by Wootters [51]:

(16) $$\begin{array}{c} C\left(\rho_{red}\right)=\max\left[0,\varepsilon_{1}-\varepsilon_{2}-\varepsilon_{3}-\varepsilon_{4}\right], \end{array}$$

where $\varepsilon_{i}$ are the square roots of the eigenvalues, in decreasing order, of the non-Hermitian matrix

(17) $$\begin{array}{c} R=\rho_{red}{\tilde{\rho }}_{red}, \end{array}$$

where $\rho_{red}$ is the matrix representation of the reduced density operator $\rho_{red}$ and ${\tilde{\rho }}_{red}$ is its corresponding spin-flipped density matrix:

(18) $$\begin{array}{c} {\tilde{\rho }}_{red}=\left(\sigma_{y}⊗\sigma_{y}\right)\rho_{red}^{*}\left(\sigma_{y}⊗\sigma_{y}\right). \end{array}$$

and $\sigma_{y}$ is the *y* component of the Pauli matrices. The concurrence values range from 0 to 1, where a zero concurrence indicates no entanglement and a concurrence value of 1 indicates maximal entanglement. The concurrence satisfies the axioms introduced by Vedral et al. [52], which define what constitutes a good entanglement measure. Other interesting measures study general quantum correlations, such as the quantum discord [53,54], which may assume a non-zero value when the entanglement vanishes. Although we do not study such measures in the current work, they are worth exploring in future research. In addition, the literature has also established measures to quantify coherence [55,56] in systems where environmental effects pose a major obstacle against preserving it. Applying such a measure to the system considered here is also worth further investigation in future works.

Throughout this work, the system parameters (*t*, Ω, $\omega_{2}$, $\lambda_{i}$, and $\Gamma_{i}$) are written in units of $\omega_{1}$ ($\equiv \omega_{1}=1$). Additionally, in what follows, the results are obtained by numerically diagonalizing the tetrahedral matrix representing the Liouville superoperator.

## 3. Entanglement Death and Revival Variation in the Presence of a Radiation Field

In the current section, we focus on the interplay between the internal parameters of the system in the presence of dephasing environments. The system will be tested at different degrees of anisotropy. The environments’ parameters are fixed to clearly demonstrate the changes instigated by introducing the field. We explore the effect of the radiation field on the entanglement dynamics by varying the field’s intensity, its coupling to the atoms, and its detuning from their natural transition frequencies. Thus, unless mentioned otherwise, the dephasing rates throughout this section are fixed to $\Gamma_{0}=0.06$, $\Gamma_{1}=0.1$, $\Gamma_{2}=0.01$ for simplicity. The effect of varying the environments’ parameters and their interplay with the radiation field is presented in the next section.

**Figure 1:**
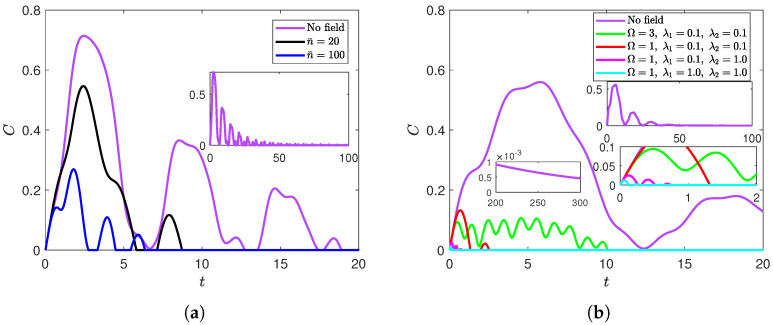
Dynamics of entanglement of the XYZ model starting from the disentangled state ${|\psi \rangle }_{D}$ at (**a**) $\Omega =\omega_{2}=3$ and $\lambda_{1}=\lambda_{2}=0.1$ for various values of $\bar{n}$; (**b**) $\omega_{2}=1$ and $\bar{n}=100$ (except for the magenta line) for various values of Ω, $\lambda_{1}$, and $\lambda_{2}$.

In Figure 1, we investigate the impact of applying a radiation field on the entanglement dynamics of a partially anisotropic system that was initially in the disentangled state ${|\psi \rangle }_{D}$. In Figure 1a, both qubits are symmetrically coupled to a radiation field of frequency Ω=3 with coupling strengths $\lambda_{1}=\lambda_{2}=0.1$. In the absence of a radiation field, the qubits suffered an asymptotic decay in entanglement content while oscillating indefinitely between death and revival states. Nevertheless, the figure illustrates that the introduction of the radiation field to the system causes stronger disentanglement effects. This is evident in the decrease in entanglement content and appearance of a terminal ESD state (no further revival) at $\bar{n}=20$. Furthermore, those disentanglement effects became stronger as the field’s intensity was increased. In particular, comparing the $\bar{n}=100$ curve with the $\bar{n}=20$ curve shows further decrease in entanglement content and a more rapid indefinite disentanglement (terminal ESD). This effect of the radiation field can be attributed to the entanglement sharing among more parties of the composite system, not only the two qubits, where part of the bipartite entanglement is reassigned to be between the qubits and the field as well as to the tripartite entanglement among the three parties [7]. This redistribution effect significantly reduces the entanglement between the two qubits. As will be shown, in all the cases we tested, this combined influence of the radiation field and the dephasing environment always leads to a complete indefinite loss of entanglement in a finite time and without further revivals.

As for the field’s frequency mode and its relation to the qubits’ transition frequencies, setting the atoms closer to resonance with the field accelerates the terminal disentanglement introduced by the field. For example, at $\bar{n}=100$ and $\lambda_{1}=\lambda_{2}=0.1$, the following three frequency settings are ordered in decreasing disentanglement time: (1) None of the atoms is at resonance with the field ($\Omega =3\neq \omega_{2}=\omega_{1}$, green line in Figure 1b). (2) One of the atoms is at resonance with the field ($\Omega =\omega_{2}=3\neq \omega_{1}$, blue line in Figure 1a). (3) Both atoms are at resonance ($\Omega =\omega_{2}=\omega_{1}$, red line in Figure 1b). In addition, Figure 1b shows a severe drop in the entanglement content and rapid acceleration of the terminal death time as the atoms interact more strongly with the field. To summarize, the field weakens the entanglement content between the qubits and kills it indefinitely in a finite time. This entanglement weakening is exacerbated when the field is more intense or interacts more strongly with the atoms. Those effects, as well as setting the field closer to resonance with one or both atoms, accelerate the final disentanglement.

**Figure 2:**
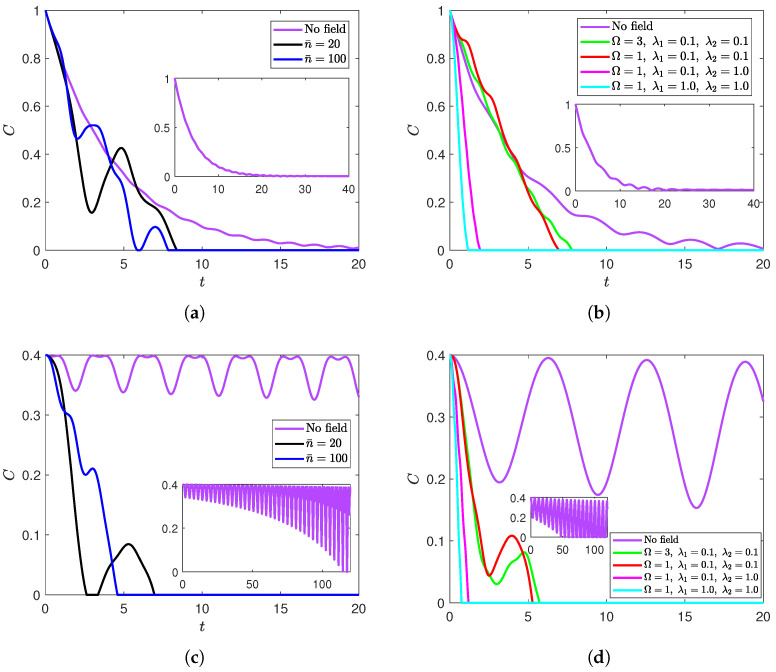
Dynamics of entanglement of the XYZ model starting from (**a**,**b**) the correlated Bell state ${|\psi \rangle }_{Bc}$ and (**c**,**d**) the Werner mixed state $\rho_{Wr}$ at (**a**–**c**) $\Omega =\omega_{2}=3$ and $\lambda_{1}=\lambda_{2}=0.1$ for various values of $\bar{n}$; (**b**–**d**) $\omega_{2}=1$ and $\bar{n}=100$ (except for the magenta line) for various values of Ω, $\lambda_{1}$, and $\lambda_{2}$.

Figure 2 demonstrates the system’s behavior under the same set of parameters of Figure 1 starting from the initial states ${|\psi \rangle }_{Bc}$ (Figure 2a,b) and $\rho_{Wr}$ (Figure 2c,d). For both initial states, the field can still be seen to cause a finite-time indefinite disentanglement. In addition, earlier observations regarding the effect of the strength of atom–field interactions on the entanglement dynamics (diminishing its content and accelerating final death as they increase) can be seen to hold for the new two states. Similarly, increasing the field’s intensity continues to shorten the final ESD time in the states ${|\psi \rangle }_{D}$ and $\rho_{Wr}$. However, increasing the intensity did not yield a straightforward behavior when it came to the scale of the entanglement values. Additionally, resonance did not exhibit a behavior as systematic as that in the disentangled state ${|\psi \rangle }_{D}$. For example, for the correlated Bell initial state (${|\psi \rangle }_{Bc}$), having one of the atoms at resonance with the field did not result in an acceleration of the final disentanglement time relative to when both atoms are out of resonance (Figure 2a,b). The Werner state, on the other hand, had a longer terminal disentanglement time, when both atoms were in resonance relative to when one of them only was at resonance. It is noteworthy that, unlike the remaining states, the correlated Bell initial state did not suffer any exact disentanglement in the absence of the radiation field, even with the influence of the dephasing environment. Although the qubits’ entanglement was decaying exponentially due to the dephasing environments, the field was solely responsible for the disentanglement periods.

As mentioned in the previous section, in addition to the XYZ partially anisotropic model discussed so far, we also investigated the behavior of the entanglement dynamics when the atoms’ mutual interaction was modeled by an XXX isotropic interaction (Appendix A.1) and by an Ising interaction (Appendix A.2). While the entanglement profiles are not exactly the same, similar overall behavior with respect to key physical parameters was observed for those models. In particular, the presence of the field did introduce a sudden terminal death, and the effect of the atom–field interaction strength on the entanglement content and speed of terminal disentanglement was sustained. Akin to the XYZ model, the effects of resonance on the entanglement dynamics showed sensitivity to the choice of initial state in the XXX and Ising models. In addition, even in the XXX and Ising models, the correlated Bell state only suffered entanglement death when the field was introduced. The terminal disentanglement time did show variation from one anisotropy model to another, but this variation was sensitive to the choice of initial state and other system parameters. We note, however, that the correlated Bell state in the Ising model exhibited a delay in disentanglement as the field’s intensity was increased, contrary to all the other cases in this work.

## 4. Entanglement Dynamics Under Varying Dephasing Rates

In this section, we turn to exploring the influence of changing the environments’ dephasing rates. More importantly, we examine how the presence of a radiation field affects the influence of the dephasing environments on the entanglement dynamics. In the previous section, the effects of the radiation field were inspected by turning the field “on” and “off” (through varying its intensity) under fixed environmental influences. Here, we examine the field impact by inspecting how strengthening the atom–field interactions and getting the qubits closer to resonance with the field will alter the response of the system to the dephasing effects. In addition, we build on our previous work [12] where we showed that strengthening the dephasing effects of the correlated environment can increase the system’s entanglement content in the absence of a radiation field. The work was an important instance of noise-enhanced efficiency [40] in environments other than the thermal bath. Here, we will demonstrate how the radiation field may enable or restrict such enhancement. To keep the work sizable and avoid prolonging the discussion, we confine the analysis to the XYZ model, as it captures the main aspects of the system dynamics.

**Figure 3:**
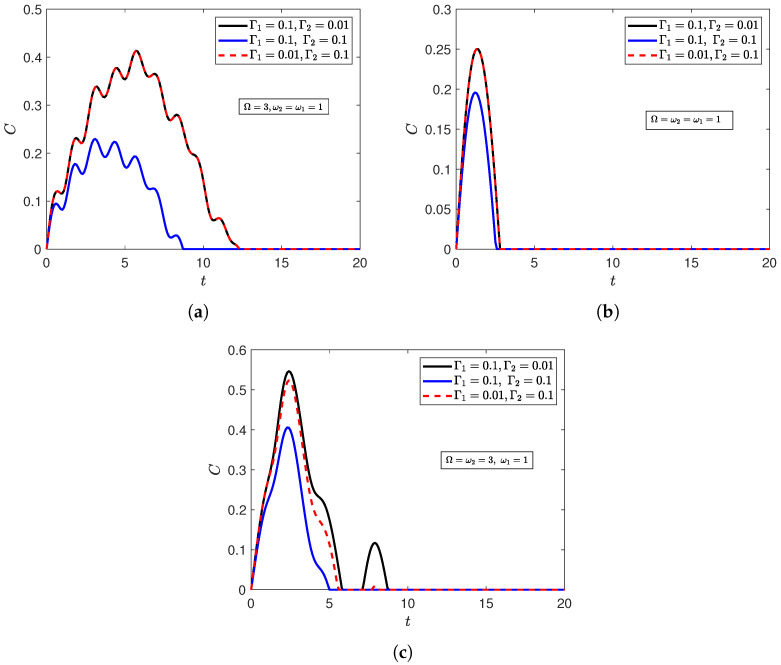
Dynamics of entanglement of the XYZ model of qubits with symmetrical atom–field interaction strengths ($\lambda_{1}=\lambda_{2}=0.1$) starting from the disentangled state ${|\psi \rangle }_{D}$ at $\bar{n}=20$, $\Gamma_{0}=0.06$, and $\left(\Gamma_{1},\Gamma_{2}\right)=\left(0.1,0.01\right),\left(0.1,0.1\right),\left(0.01,0.1\right)$ when (**a**) both qubits are out of resonance with the field ($\Omega =3,\omega_{2}=1$); (**b**) both qubits are in resonance ($\Omega =\omega_{2}=1$); (**c**) one qubit is in resonance with the field ($\Omega =1,\omega_{2}=3$).

In Figure 3, we assume that the qubits are symmetrically interacting with the field ($\lambda_{1}=\lambda_{2}$), and examine the interplay between their detuning from the field’s frequency mode and the influence of the independent dephasing environments. Overall, the figure shows that strengthening the dephasing rates on both environments weakens the entanglement between the qubits. The effect is shown to be exacerbated when both qubits are out of resonance with the field (Figure 3a). In other words, getting the qubits close to resonance with the cavity’s radiation field (which is not directly interacting with the environments) shields them from the dephasing effects of the independent environments. We note that when the qubits are symmetric (Figure 3a,b), interchanging the asymmetric dephasing rates has no effect on the entanglement dynamics. This behavior is anticipated for symmetric initial states, since entanglement is a shared property of the two qubits and should show no bias towards either qubit parameters. However, this will not hold for the Werner state, for it is not symmetric (Equation (13)). The corresponding curves ($\left(\Gamma_{1},\Gamma_{2}\right)=\left(0.1,0.01\right)$, and (0.01,0.1)) are included for consistency with Figure 3c, and to serve as a check for the validity of the mathematical formulations and the numerical calculations carried out in this work. Nevertheless, when the qubits are asymmetric (Figure 3c), setting the qubit suffering stronger dephasing effects to resonance ($\Gamma_{2}=10\Gamma_{1}⟶\omega_{2}=\Omega$) diminishes the system’s entanglement content, and sends the system to a terminally disentangled state more rapidly. This suggests that the environment has a stronger effect on the system, overall, when the dephasing effects impact the qubit that is closer to resonance with the field.

**Figure 4:**
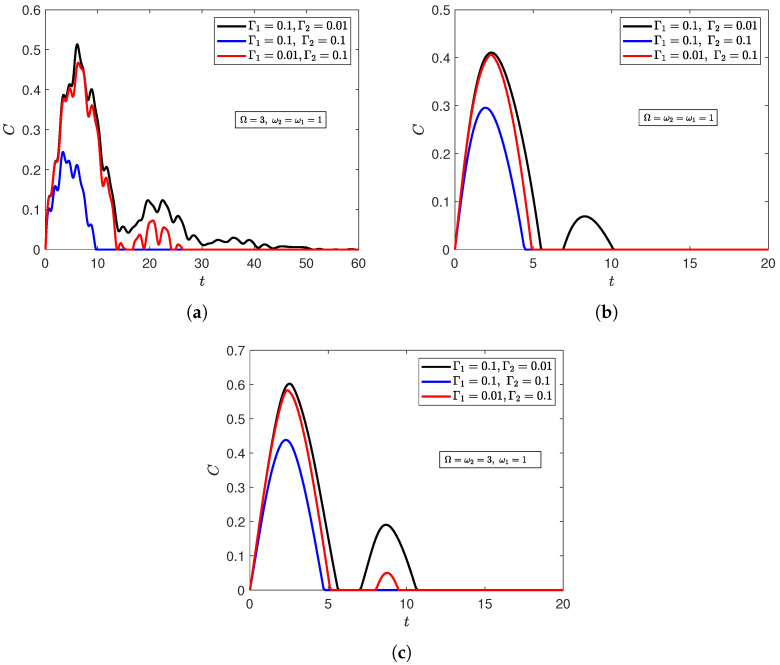
Dynamics of entanglement of the XYZ model of qubits with asymmetrical atom–field interaction strengths ($\lambda_{1}=0.01,\lambda_{2}=0.1$) starting from the disentangled state ${|\psi \rangle }_{D}$ at $\bar{n}=20$, $\Gamma_{0}=0.06$, and $\left(\Gamma_{1},\Gamma_{2}\right)=\left(0.1,0.01\right),\left(0.1,0.1\right),\left(0.01,0.1\right)$ when (**a**) both qubits are out of resonance with the field ($\Omega =3,\omega_{2}=1$); (**b**) both qubits are in resonance ($\Omega =\omega_{2}=1$); (**c**) one qubit is in resonance with the field ($\Omega =1,\omega_{2}=3$).

To investigate the interplay between $\lambda_{i}$ and $\Gamma_{i}$, Figure 4 demonstrates the corresponding dynamics of Figure 3 but for asymmetrical interaction strengths ($\lambda_{1}\neq \lambda_{2}$). The aforementioned effects in Figure 3 can all be seen to take place in Figure 4, i.e., the effect of strengthening the dephasing rates of both qubits and setting qubits closer/further from resonance with the field. However, Figure 4 shows that if the qubit that is strongly interacting with the field ($\lambda_{2}$) suffers greater dephasing effects ($\left(\Gamma_{1},\Gamma_{2}\right)=\left(0.01,0.1\right)$), then the corresponding independent dephasing environment has a greater capability of diminishing the entanglement content and speeding up terminal disentanglement. Linking the current section with the previous one, a comparison between Figure 3 and Figure 4 shows that increasing interaction of one qubit with the field can significantly reduce the entanglement between the qubits. This is evident in the decrease in entanglement content of all curves in Figure 3 relative to their counterparts in Figure 4. We investigated the corresponding effects of qubits’ detuning and atom–field interaction strength for a system starting from the Bell initial state (Appendices Appendix B.1.1 and Appendix B.1.2), and the same behaviors were observed. However, remarkably, the Werner initial state (Appendices Appendix B.2.1 and Appendix B.2.2) showed an increase in entanglement when the atom that strongly interacted with the field was set to resonance and endured a stronger dephasing rate (Appendix B.2.2).

**Figure 5:**
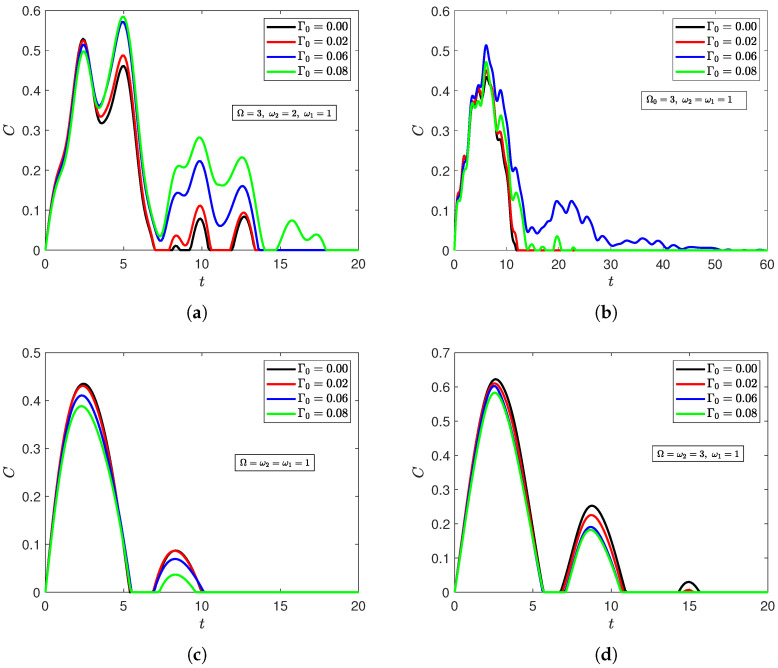
Dynamics of entanglement of the XYZ model of qubits with asymmetrical atom–field interaction strengths ($\lambda_{1}=0.01,\lambda_{2}=0.1$) starting from the disentangled state ${|\psi \rangle }_{D}$ at $\bar{n}=20$, $\Gamma_{1}=0.1$, and $\Gamma_{2}=0.01$, and $\Gamma_{0}=0.00,0.02,0.06,0.08$ when (**a**) both qubits are asymmetrically out of resonance with the field ($\Omega =3,\omega_{2}=2$); (**b**) both qubits are symmetrically out of resonance with the field ($\Omega =3,\omega_{2}=1$); (**c**) both qubits are in resonance ($\Omega =\omega_{2}=1$); (**d**) one qubit is at resonance with the field ($\Omega =\omega_{2}=3$).

**Figure A5:**
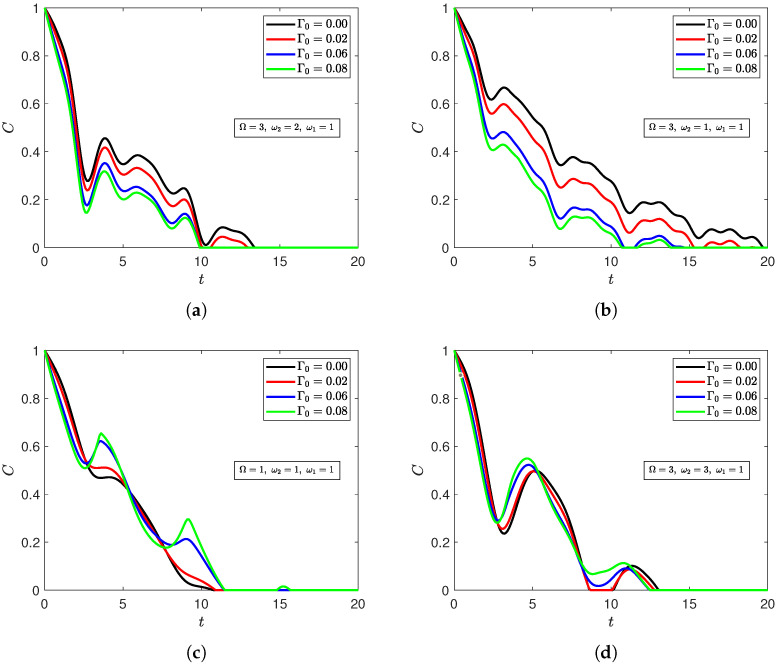
Dynamics of entanglement of the XYZ model of qubits with asymmetrical atom–field interaction strengths ($\lambda_{1}=0.01,\lambda_{2}=0.1$) starting from the correlated Bell state ${|\psi \rangle }_{Bc}$ at $\bar{n}=20$, $\Gamma_{1}=0.1$, and $\Gamma_{2}=0.01$, and $\Gamma_{0}=0.00,0.02,0.06,0.08$ when (**a**) both qubits are asymmetrical out of resonance with the field ($\Omega =3,\omega_{2}=2$); (**b**) both qubits are symmetrical out of resonance with the field ($\Omega =3,\omega_{2}=1$); (**c**) both qubits are in resonance ($\Omega =\omega_{2}=1$); (**d**) one qubit is at resonance with the field ($\Omega =\omega_{2}=3$).

Figure 5 investigates the effects of the introduced radiation field on noise-enhanced efficiency for the initially disentangled state ${|\psi \rangle }_{D}$. The figure shows that noise-enhanced efficiency can still occur when the qubits are trapped with a cavity radiation field. However, this was limited to scenarios when the qubits were out of resonance with the radiation field, whether asymmetrically (Figure 5b) or symmetrically (Figure 5a). Once either qubit was at resonance with the field, the entanglement profile was completely transformed, and strengthening the correlated dephasing environment turned to diminish the entanglement content instead of empowering it (Figure 5c,d). Interestingly, the radiation field had the exact opposite effect on the correlated Bell initial state ${|\psi \rangle }_{Bc}$ (Appendix B.1.3). In particular, a greater dephasing rate of the correlated environment only increases the entanglement content when one of the qubits was set at resonance with the field (Figure A5c,d), and weakens the entanglement content when both qubits were out of resonance (asymmetrically in Figure A5a and symmetrically in Figure A5b). The Werner state was also investigated, and it exhibited a noise-enhanced efficiency regardless of the atoms’ detuning from the field (Appendix B.2.3). Those results illustrate that the field can play an important role in controlling the effect of the correlated dephasing environment on the two-qubit system, but one that is also highly sensitive to the system’s initial state. The results also demonstrate the lack of a straightforward relationship between resonance and noise-enhanced efficiency due to strong dependence on the initial state.

## 5. Conclusions

We investigated a system of two asymmetric interacting qubits coupled to independent and dependent dephasing environments. The two qubits enjoy a general XYZ spin-1/2 interaction and are coupled to a common coherent radiation field. We studied the system dynamics by numerically diagonalizing the Liouville superoperator corresponding to the Lindblad master equation and obtaining the time-dependent density operator and concurrence. We then studied the effects of the cavity’s radiation on the concurrence over ranges of the system internal parameters and initial states and for three different degrees of anisotropy; the XYZ partially anisotropic system, the Ising system, and the XXX isotropic system. We demonstrated the field’s ability to cause rapid death in finite time and prevent any future revival of entanglement. The field also diminished the entanglement content of the two-qubit system. Those effects were shown to be exacerbated when the atom–field interaction strength was increased. However, the effect of other system parameters, such as the qubits’ detunings and the field’s intensity, showed severe sensitivity to the choice of initial state. Additionally, we showed that, for some initial states, bringing the atoms’ transition frequencies closer to resonance empowers the effects of the independent dephasing environments. For those states, we showed that strengthening the independent dephasing rate on a qubit with a strong atom–field interaction would result in further reduction in the entanglement content than on a qubit with a weak atom–field interaction. Finally, we investigated if the correlated dephasing environment can induce a noise-enhanced efficiency while the qubits are interacting with an external radiation field. We illustrated that the radiation field can have a strong influence on whether such enhancement would occur or not, but in a manner that is highly sensitive to the qubits’ initial state. We also showed that this sensitivity to the initial state prevents a straightforward relationship between the noise-enhanced efficiency and the qubits’ detunings from the field.

## Figures and Tables

**Figure A1:**
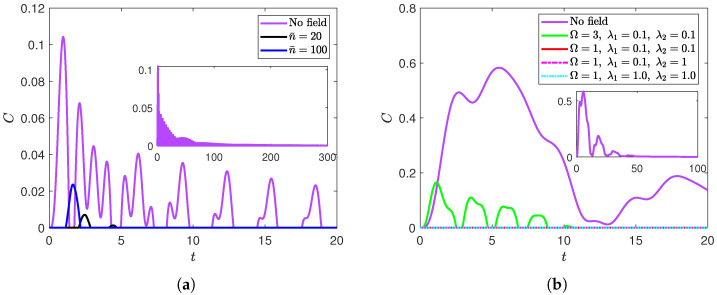

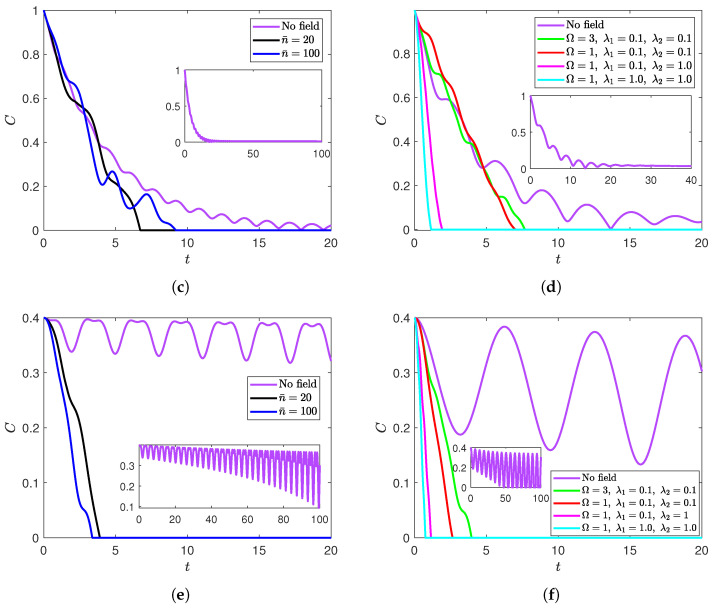
Dynamics of entanglement of the Ising model starting from (**a**,**b**) the disentangled state ${|\psi \rangle }_{D}$, (**c**,**d**) the disentangled state ${|\psi \rangle }_{Bc}$, and (**e**,**f**) the Werner mixed state $\rho_{Wr}$ at (**a**,**c**,**e**) $\Omega =\omega_{2}=3$ and $\lambda_{1}=\lambda_{2}=0.1$ for various values of $\bar{n}$; (**b**,**d**,**f**) $\omega_{2}=1$ and $\bar{n}=100$ (except for the magenta line) for various values of Ω, $\lambda_{1}$, and $\lambda_{2}$.

**Figure A2:**
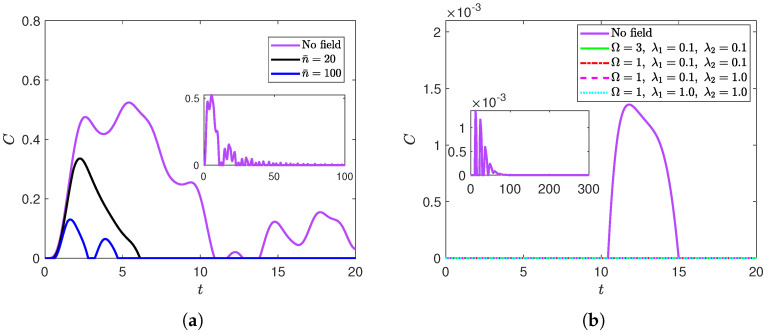

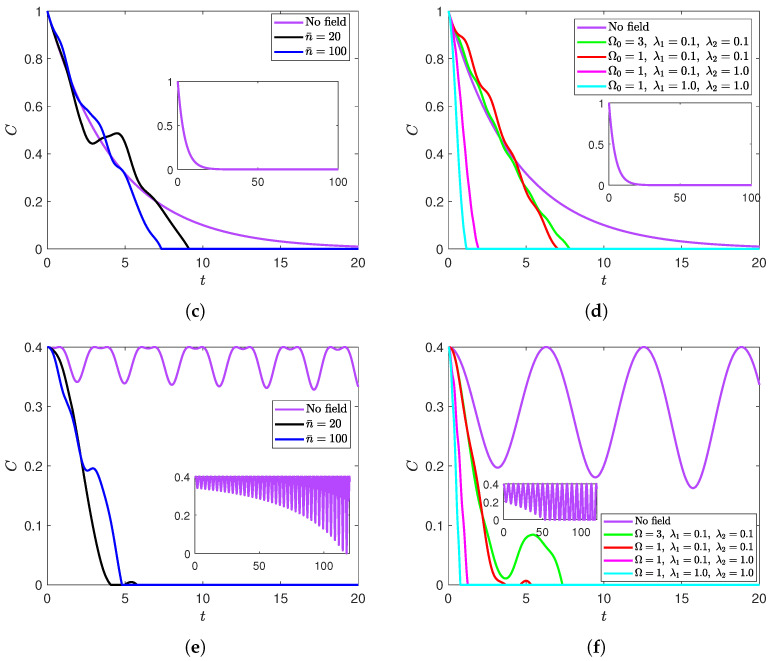
Dynamics of entanglement of the XXX model starting from (**a**,**b**) the disentangled state ${|\psi \rangle }_{D}$, (**c**,**d**) the disentangled state ${|\psi \rangle }_{Bc}$, and (**e**,**f**) the Werner mixed state $\rho_{Wr}$ at (**a**,**c**,**e**) $\Omega =\omega_{2}=3$ and $\lambda_{1}=\lambda_{2}=0.1$ for various values of $\bar{n}$; (**b**,**d**,**f**) $\omega_{2}=1$ and $\bar{n}=100$ (except for the magenta line) for various values of Ω, $\lambda_{1}$, and $\lambda_{2}$.

**Figure A3:**
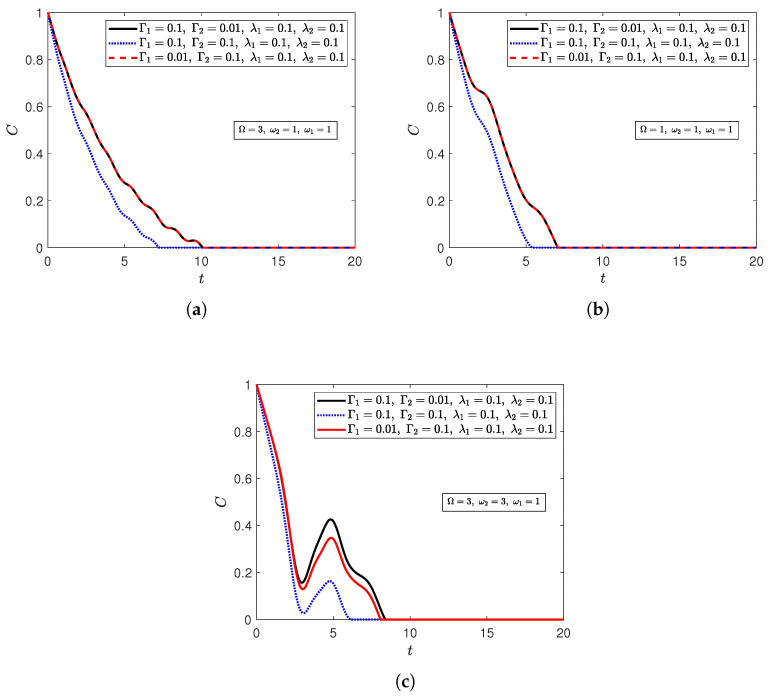
Dynamics of entanglement of the XYZ model of qubits with symmetrical atom–field interaction strengths ($\lambda_{1}=\lambda_{2}=0.1$) starting from the disentangled state ${|\psi \rangle }_{Bc}$ at $\bar{n}=20$, $\Gamma_{0}=0.06$, and $\left(\Gamma_{1},\Gamma_{2}\right)=\left(0.1,0.01\right),\left(0.1,0.1\right),\left(0.01,0.1\right)$ when (**a**) both qubits are out of resonance with the field ($\Omega =3,\omega_{2}=1$); (**b**) both qubits are in resonance ($\Omega =\omega_{2}=1$); (**c**) one qubit is in resonance with the field ($\Omega =1,\omega_{2}=3$).

**Figure A4:**
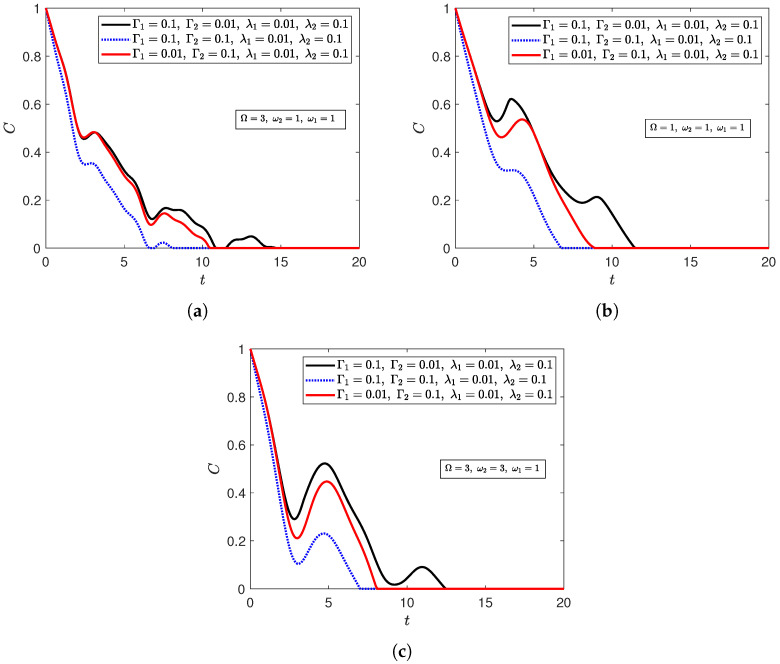
Dynamics of entanglement of the XYZ model of qubits with asymmetrical atom–field interaction strengths ($\lambda_{1}=0.01,\lambda_{2}=0.1$) starting from the disentangled state ${|\psi \rangle }_{Bc}$ at $\bar{n}=20$, $\Gamma_{0}=0.06$, and $\left(\Gamma_{1},\Gamma_{2}\right)=\left(0.1,0.01\right),\left(0.1,0.1\right),\left(0.01,0.1\right)$ when (**a**) both qubits are out of resonance with the field ($\Omega =3,\omega_{2}=1$); (**b**) both qubits are in resonance ($\Omega =\omega_{2}=1$); (**c**) one qubit is in resonance with the field ($\Omega =1,\omega_{2}=3$).

**Figure A6:**
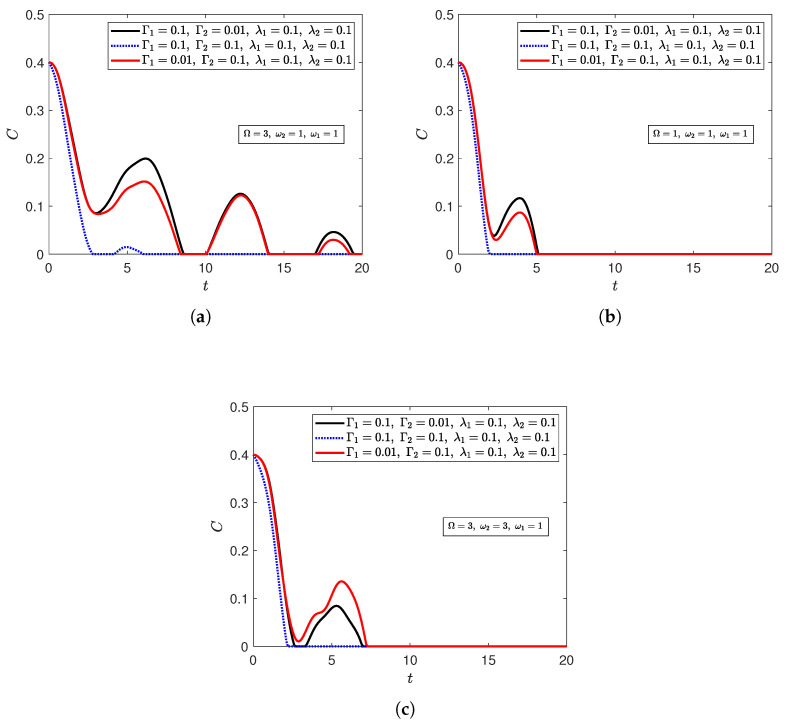
Dynamics of entanglement of the XYZ model of qubits with symmetrical atom–field interaction strengths ($\lambda_{1}=\lambda_{2}=0.1$) starting from the disentangled state $\rho_{Wr}$ at $\bar{n}=20$, $\Gamma_{0}=0.06$, and $\left(\Gamma_{1},\Gamma_{2}\right)=\left(0.1,0.01\right),\left(0.1,0.1\right),\left(0.01,0.1\right)$ when (**a**) both qubits are out of resonance with the field ($\Omega =3,\omega_{2}=1$); (**b**) both qubits are in resonance ($\Omega =\omega_{2}=1$); (**c**) one qubit is in resonance with the field ($\Omega =1,\omega_{2}=3$).

**Figure A7:**
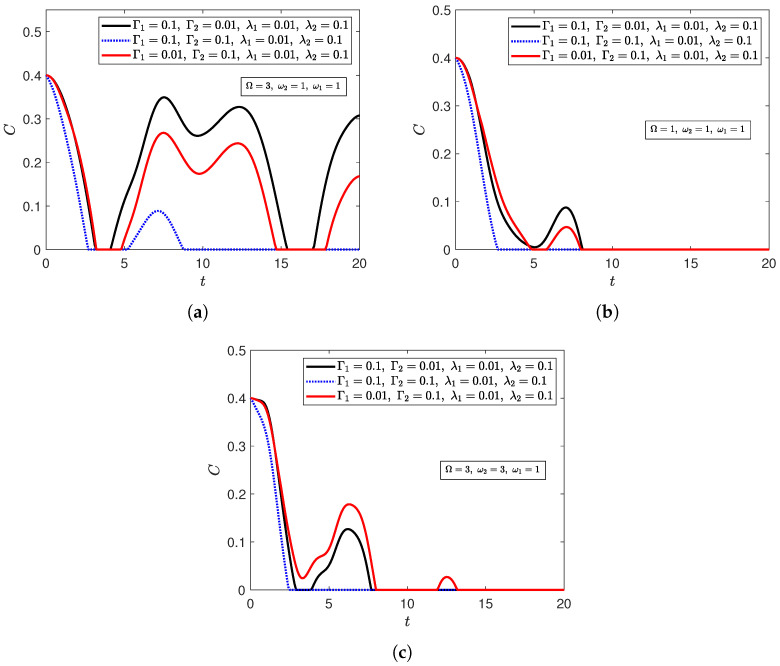
Dynamics of entanglement of the XYZ model of qubits with asymmetrical atom–field interaction strengths ($\lambda_{1}=0.01,\lambda_{2}=0.1$) starting from the disentangled state $\rho_{Wr}$ at $\bar{n}=20$, $\Gamma_{0}=0.06$, and $\left(\Gamma_{1},\Gamma_{2}\right)=\left(0.1,0.01\right),\left(0.1,0.1\right),\left(0.01,0.1\right)$ when (**a**) both qubits are out of resonance with the field ($\Omega =3,\omega_{2}=1$); (**b**) both qubits are in resonance ($\Omega =\omega_{2}=1$); (**c**) one qubit is in resonance with the field ($\Omega =1,\omega_{2}=3$).

**Figure A8:**
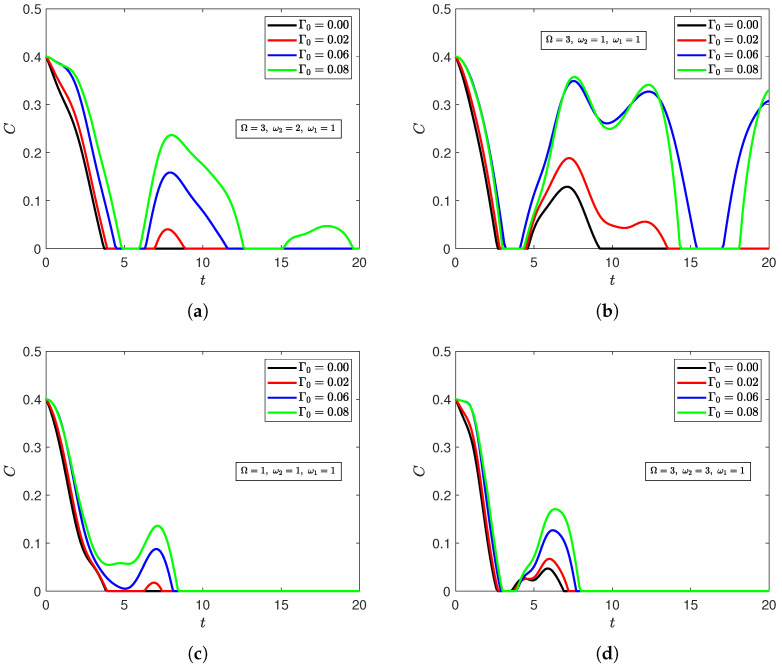
Dynamics of entanglement of the XYZ model of qubits with asymmetrical atom–field interaction strengths ($\lambda_{1}=0.01,\lambda_{2}=0.1$) starting from the correlated Bell state $\rho_{Wr}$ at $\bar{n}=20$, $\Gamma_{1}=0.1$, and $\Gamma_{2}=0.01$, and $\Gamma_{0}=0.00,0.02,0.06,0.08$ when (**a**) both qubits are asymmetrical out of resonance with the field ($\Omega =3,\omega_{2}=2$) (**b**) both qubits are symmetrical out of resonance with the field ($\Omega =3,\omega_{2}=1$); (**c**) both qubits are in resonance ($\Omega =\omega_{2}=1$); (**d**) one qubit is at resonance with the field ($\Omega =\omega_{2}=3$).

## Data Availability

The data presented in this study are contained within the article.

## Abbreviations

The following abbreviations are used in this manuscript:

QIP	Quantum Information Processing
ESD	Entanglement Sudden Death
ESB	Entanglement Sudden Birth

## Appendix A

The current appendix illustrates how introducing the cavity radiation field alters the behavior of the time-evolving dynamics of entanglement for the Ising and XXX interaction models and starting from the initial states ${|\psi \rangle }_{D}$, ${|\psi \rangle }_{Bc}$, and $\rho_{Wr}$. This is done by demonstrating the results corresponding to Figure 1 and Figure 2, but for the Ising (Appendix A.1) and XXX (Appendix A.2) models.

## Appendix B

The current appendix illustrates how the influence of dephasing environments on the qubits’ entanglement dyanamics is affected by the presence of a radiation field for the Ising and XXX interaction models and starting from the initial states ${|\psi \rangle }_{D}$, ${|\psi \rangle }_{Bc}$, and $\rho_{Wr}$. This is done by demonstrating the results corresponding to Figure 3, Figure 4 and Figure 5, but for the Ising (Appendix B.1) and XXX (Appendix B.2) models.
